# Simulating the drug discovery pipeline: a Monte Carlo approach

**DOI:** 10.1186/1758-2946-4-32

**Published:** 2012-11-27

**Authors:** Melvin J Yu

**Affiliations:** 1 Eisai Inc., 4 Corporate Dr., Andover, MA, 01810, USA

## Abstract

**Background:**

The early drug discovery phase in pharmaceutical research and development marks the beginning of a long, complex and costly process of bringing a new molecular entity to market. As such, it plays a critical role in helping to maintain a robust downstream clinical development pipeline. Despite its importance, however, to our knowledge there are no published in silico models to simulate the progression of discrete virtual projects through a discovery milestone system.

**Results:**

Multiple variables were tested and their impact on productivity metrics examined. Simulations predict that there is an optimum number of scientists for a given drug discovery portfolio, beyond which output in the form of preclinical candidates per year will remain flat. The model further predicts that the frequency of compounds to successfully pass the candidate selection milestone as a function of time will be irregular, with projects entering preclinical development in clusters marked by periods of low apparent productivity.

**Conclusions:**

The model may be useful as a tool to facilitate analysis of historical growth and achievement over time, help gauge current working group progress against future performance expectations, and provide the basis for dialogue regarding working group best practices and resource deployment strategies.

## Background

Over the past fifteen years, productivity of pharmaceutical research and development (R&D) in terms of launched new molecular entities (NME) has at best been flat while costs have risen, resulting in a sharp decline in the number of first-in-class drugs entering the market as a percentage of R&D expenditures [1,2]. Adoption of new technologies, six sigma initiatives [3,4] and adaptive clinical trial designs [5,6] have led to incremental improvements, but the productivity challenge facing the pharmaceutical industry as a whole remains. Although studies in this area largely focus on the clinical development aspect of the pipeline, a steady supply of quality preclinical drug candidates must be maintained at the front end to prevent downstream gaps from forming. For example, shifting the focus from late stage phase III studies to early clinical proof of concept (POC) would require an abundance of preclinical candidate compounds that both add projected value to the company’s portfolio and have a reasonable probability of technical success.

**Figure 1:**
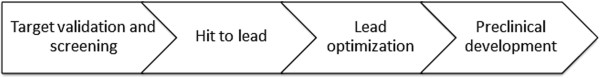
**Diagram of major milestone transition and go/no-go decision points in the discovery phase of pharmaceutical R&D leading to preclinical development.** Details as follows: (1) Conduct exploratory work that includes project conception, target validation and screening. (2) Identify chemical lead(s) and establish target drug profile. (3) Optimize chemical properties and biological activity against the target drug profile. (4) Complete all necessary studies to allow an investigational new drug (IND) application to be filed.

The study by Paul and co-workers^1^ indicates that the lead optimization phase of drug discovery contributes significantly toward the out-of-pocket cost per launch of an NME. Yet, despite the importance of the discovery phase in pharmaceutical R&D, studies are lacking in the literature to provide guidance regarding the optimal distribution of scientific resources to generate a sufficient quantity of preclinical candidate compounds at a rate that would lead to an NME entering the marketplace in a specific time-frame. While particular sub-milestones and milestone names may vary between the major pharmaceutical companies, the primary transition and go/no-go decision points for the early drug discovery pipeline can be categorized as those depicted in Figure 1.

Attrition rates for compounds as they move through the milestone system have been published^1^ and could be used to approximate the average percent success rate of projects transitioning from one stage to the next. The question that we asked was whether this information could be employed to derive useful guidelines for human resource distribution by scientific discipline, and what the optimal number of chemists and biologist might be to support a given portfolio of discovery project types under a specific set of conditions.

Although a number of simulation and game theory algorithms have been used in clinical development models, [7] to our knowledge there are no published in silico models that simulate the progression of projects through early discovery, i.e. from conceptualization to candidate selection. Given the importance of the discovery phase to pharmaceutical R&D, we developed a Monte Carlo simulation algorithm for modeling discrete virtual projects as they move through the discovery milestone system ending with entry into preclinical development. Each virtual project has associated with it a dynamic number of chemists and biologists with drug metabolism/pharmacokinetics (DMPK) support that varies according to project priority, type, and milestone stage. Such an in silico model would allow multiple variables to be evaluated for a given portfolio of drug discovery programs in the context of expected productivity metrics.

## Methods

With scientific specialization comes the need for cross-functional teams [8]. Pharmaceutical R&D is no exception. For purposes of the model, however, a simplifying assumption is made that drug discovery project teams consist of medicinal/synthetic chemists, biologists capable of developing and running multi-tiered in vitro assay systems and relevant in vivo animal models as well as representation by members of the DMPK group. While scientists from a number of specialized scientific disciplines (e.g. process chemistry research, analytical chemistry, computational chemistry, biopharmaceutical assessment, formulation, in vivo imaging, etc.) participate to varying degrees in the drug discovery process, their roles are assumed not to be rate limiting with regard to decision-making until projects approach the preclinical development go/no-go decision point. Cost and other considerations often limit, for example, either preclinical animal multispecies allometric scaling or in silico simulations that use experimental in vivo and in vitro data to derive human drug exposure and dose projection values. Since these studies are usually, if not exclusively, performed on candidate compounds and not during the early phase of drug discovery, we assume for purposes of the model that chemistry, biology and discovery DMPK represent the rate limiting scientific disciplines during early discovery, depending on the scientific issues to be addressed. Although data generated by the other specialized disciplines are certainly used by project teams to help guide a structure-activity relationship (SAR) investigation, the model assumes that there is enough resource support from the ancillary disciplines to allow decisions to be made within the discovery cycle times specified by the user.

**Figure 2:**
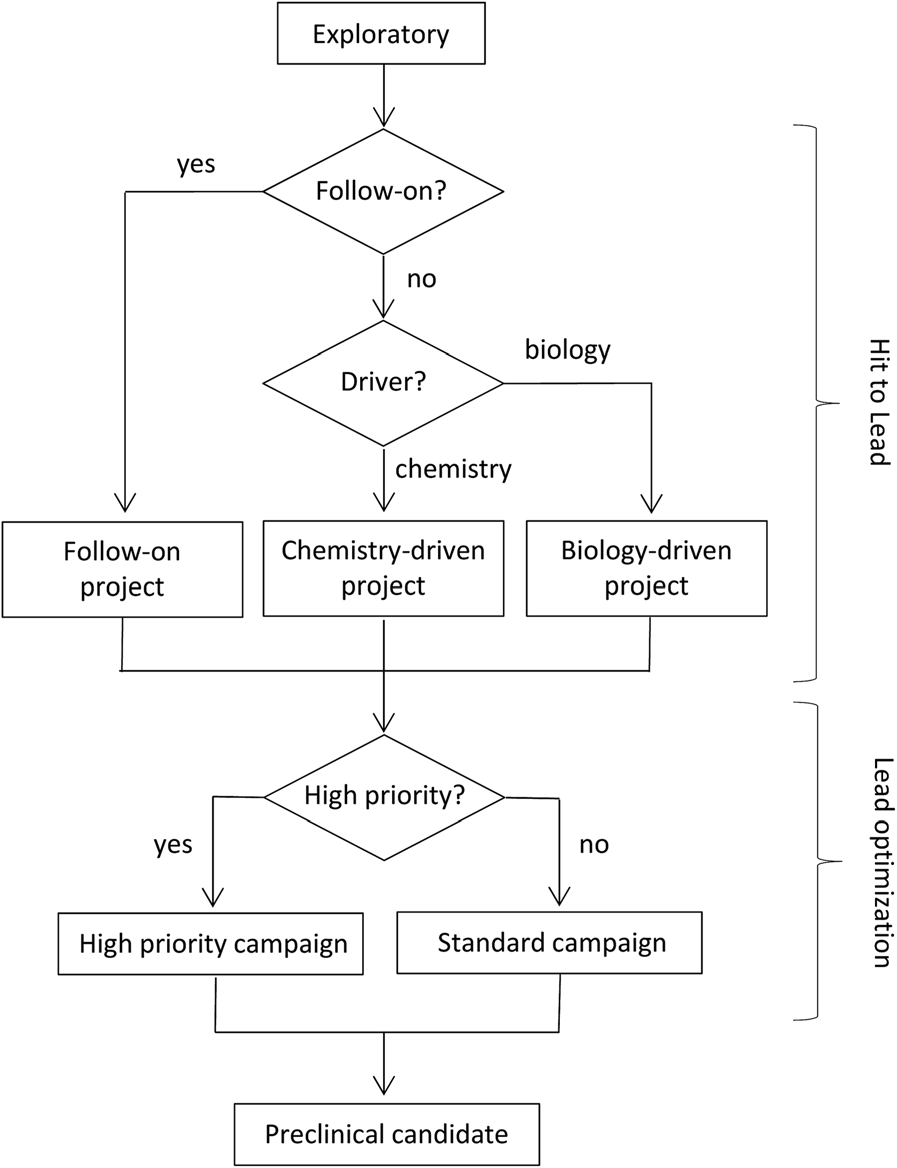
Algorithm flow chart that spans project ideation (exploratory) to candidate selection for project type (hit to lead) and priority (lead optimization).

The algorithm takes as input a series of user-defined numbers that describes a typical project at the hit to lead and lead optimization stages. This includes a target number of chemists and biologists, cycle time, milestone transition probabilities, and project type. Project type include those that are biology-driven (i.e., projects that typically involve high-throughput screening (HTS) against a biological target of interest), and those that are chemistry-driven with a clearly identified chemical lead, but not necessarily information about mechanism of action (MOA). In either case, the link between MOA and human disease may or may not be established. Another type of project are those that are “follow-on” or “back-up” projects that share biological resources and therapeutic target with another on-going discovery program, albeit with a different structural scaffold or chemical lead. In such cases, the SAR will likely diverge from its related project, and will, if successful, afford a candidate compound with a similar drug profile, but with a different scaffold and therefore a different set of physicochemical properties. The algorithm treats the two as separate and distinct projects, albeit with different levels of chemical and biological support, whose outcome could both lead to preclinical candidate compounds. The user has the ability to specify the percent of projects that are “follow-on” and the percent of those that are chemistry versus biology driven (Figure 2).

Since project cycle time is dependent on the level of resource support, a simple monotonic function was used to correct the time to milestone decision. For example, the algorithm may allow a project at the hit to lead stage to reach lead optimization in one year if fully staffed, but would correct that value to two years if only half-staffed. If additional resources become available in-between the hit to lead and lead optimization stages, then the time to milestone decision would be adjusted according to Equation 1. In this fashion, project cycle times are linearly dependent on the level of staffing up to the target number of chemists or biologists. The total number of scientists on the project team is not allowed to exceed the target value specified by the user. For hit to lead projects, the level of staffing is adjusted based on probability of success defined by the original random number assigned at the time it transitioned from exploratory. Maximum staffing levels for all hit to lead projects are calculated using Equation 2.

The full time equivalent (FTE) efficiency parameter as defined by the model is the fractional amount of time spent on direct project related activities not including, for example, recruiting, general training, scientific conferences, meetings, etc., and is a function of group size (vide infra).

(1) $$\text{A}=\frac{\text{CT}\left(\text{TC}+\text{TB}\right)}{\text{F·}\left(\text{C}+\text{B}\right)}$$

$$\text{Updated TD}=\text{A}-\frac{\text{TE·}\text{A}}{\text{old TD}}$$

Where the following definitions apply:

CT = cycle time to next milestone

TC = target no. chemists for project at current milestone

TB = target no. biologists for project at current milestone

F = FTE efficiency

C = updated no. chemists assigned to the project

B = updated no. biologists assigned to the project

TE = time elapsed since last milestone transition

TD = time to next milestone go/no-go decision

(2) $$\text{max}=\text{ceiling}\left(\text{r}\cdot \text{s}/\text{t}\right)$$

Where:

r = random number (0 ≤ r < 1) assigned at time of transition from screening (i.e., exploratory) to hit to lead

s = target no. chemists/biologists at hit to lead stage

t = threshold for successfully transitioning from exploratory to hit to lead

max = maximum number of chemists/biologists that can be assigned to this particular hit to lead project. If r = 0, then only the default number of biologists and chemists can assigned to the project.

Virtual projects are created and progress through the drug discovery pipeline by successfully passing a series of go/no-go decision points based on a random number assignment and comparison against the user specified probability of success threshold for the particular milestone transition. If the random number is below the threshold, then the project successfully transitions to the next milestone. If not, then the project is terminated and scientists are reassigned to other activities as described below. For example, if a screening project is ready to progress to hit to lead, a random number is generated. If that number is less than the threshold value for moving to the next milestone, then the project is considered to have successfully transitioned to the hit to lead phase. By default, the project is then staffed by one chemist and one biologist. The random number also represents that hit to lead project’s priority, which is then used to calculate the maximum number of additional chemists or biologists that can be added to the project (Equation 2). To illustrate the point, if the target number of hit to lead biologists is 3, the random number is 0.5 and the threshold value is 0.8, then that hit to lead project can only be staffed with a maximum of 2 biologists. In other words, only one additional biologist can be added on top of the original default number of 1. If the random number is zero, then no additional biologists can be added. In this manner, hit to lead projects are resourced commensurate with their assigned priority. Hit to lead project progression to lead optimization proceeds in a similar manner. However, if the transition is successful all attempts are made to fully staff the project as defined by the user. Projects at the lead optimization stage can also receive DMPK support, which effectively acts to accelerate SAR decision-making (and consequently compress the time to next milestone) by allowing analogues with poor in vivo exposure to be identified as early as possible. In this manner cycle times can be reduced. Implicit within the model is that milestone go/no-go decisions are rendered based on scientific merit that takes into account compound druggability and physicochemical properties.

**Figure 3:**
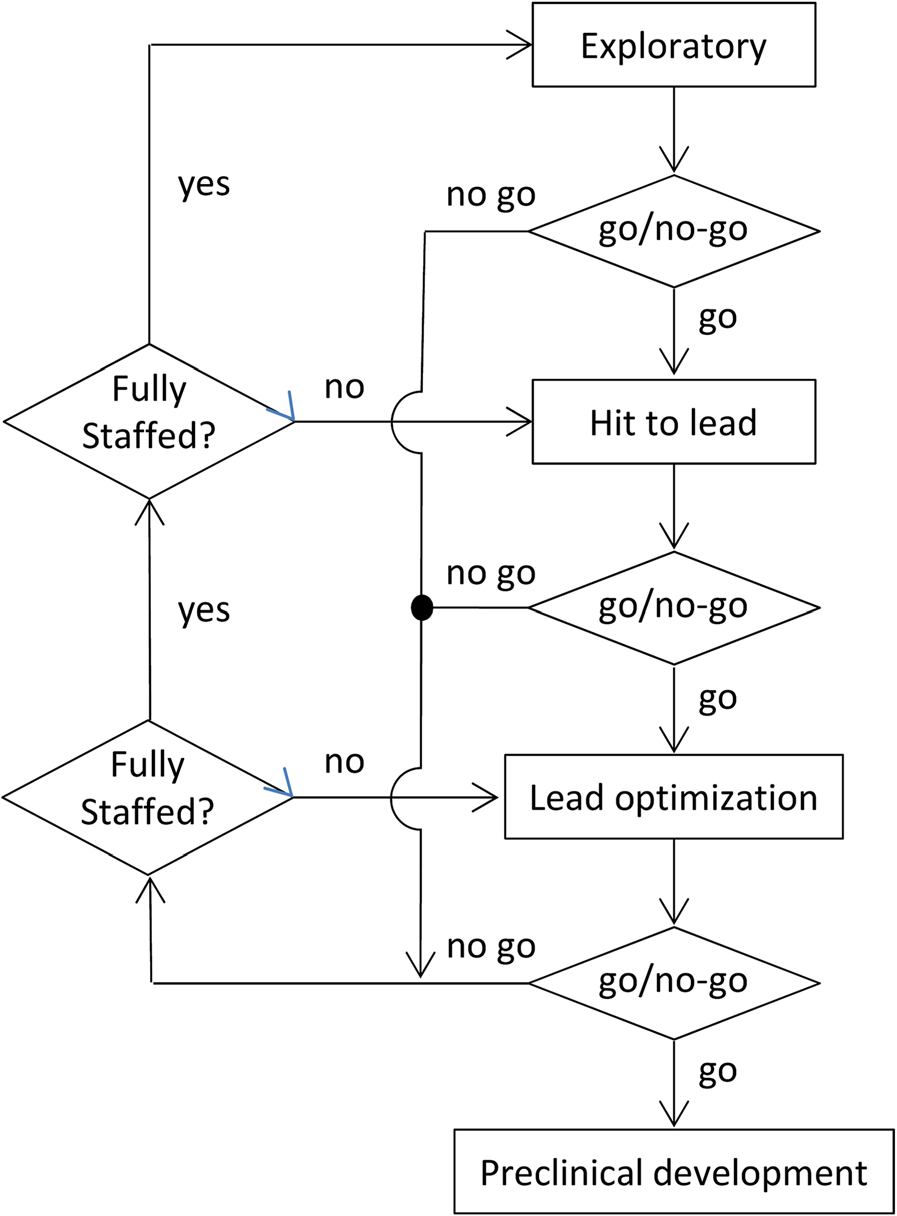
**Decision flow chart of project milestone progression and scientist reassignment priority.** Assignment of scientists to hit to lead projects is first to those on hold (if any) and second to any on-going understaffed efforts.

Scientists are released for reassignment when their project experiences the following: (a) failure to pass a milestone and is terminated, (b) temporarily put on hold to free resources for a high priority lead optimization campaign or (c) successfully enters preclinical development. The order of reassignment is first, to on-going understaffed lead optimization projects, second, to re-staff any hit to lead projects that were put on hold, third to on-going understaffed hit to lead activities, and fourth, to new project ideation and generation (exploratory projects) as outlined in Figure 3. New project activities include biological target identification, validation and compound library screening or in the case of chemistry-driven programs, identification of a chemical starting point through professional contacts (e.g. academic collaborations, strategic alliances, in-licensing, etc.), scientific conferences or the literature. Projects are considered understaffed if the number of assigned chemists and biologists is below their target values. As is true in many large pharmaceutical companies, the model assumes that all scientists spend a percentage of their time pursuing exploratory activities in addition to their primary project responsibilities. Any scientist not assigned to a specific milestone project is considered to be working on these type of activities. As a result, the model does not track discrete exploratory efforts, but rather assumes an excess of ideas relative to the number of scientists, i.e., that there are more research proposals to work on than time or resources will allow.

The algorithm allows for the successful transition of hit to lead projects into two types of lead optimization campaigns: high priority and standard priority (Figure 2). For high priority projects, the algorithm first reassigns scientists working on exploratory efforts. If fully staffing the project team is not possible, then the algorithm will selectively terminate hit to lead projects starting with those that are the least supported. If full staffing is still not possible despite terminating all hit to lead projects and diverting effort from all exploratory activities, then the project is still allowed to transition. Additional scientists are added as they become available. For standard priority lead optimization campaigns the staffing level is maintained from its hit to lead level and scientists are added to the project team as availability allows. In both instances, the time to milestone go/no-go decision is adjusted accordingly. The ratio between these two types of projects is user-defined and will depend on individual company strategy, policies and procedures.

A working group is defined by the model as an organizational structure comprised of scientists in a single cross-functional unit such as a department, division, or center that targets a specific therapeutic area or subspecialty (e.g., obesity within metabolic disorders could be considered a working group). As used by the model, the scientists within the working group support a number of different individual projects with varying degrees of staffing at different points along the drug discovery pipeline. *In other words*, *members of a working group* (*i*.*e*., *unit*, *department*, *division*, *or center* – *different companies use different names*) *work to support multiple discovery project teams*. Since there will likely be multiple working groups, each supporting multiple projects, within a large organization, the model would consider each of these units as essentially autonomous, i.e., operating largely independent of one another in a managerial and budgetary sense. Of course, some degree of communication and cooperation between the therapeutic and specialty units in a larger organization must take place for synergy and process gains to occur, and for shared experience and tacit scientific knowledge to drive the corporate knowledge creation spiral [9,10].

**Figure 4:**
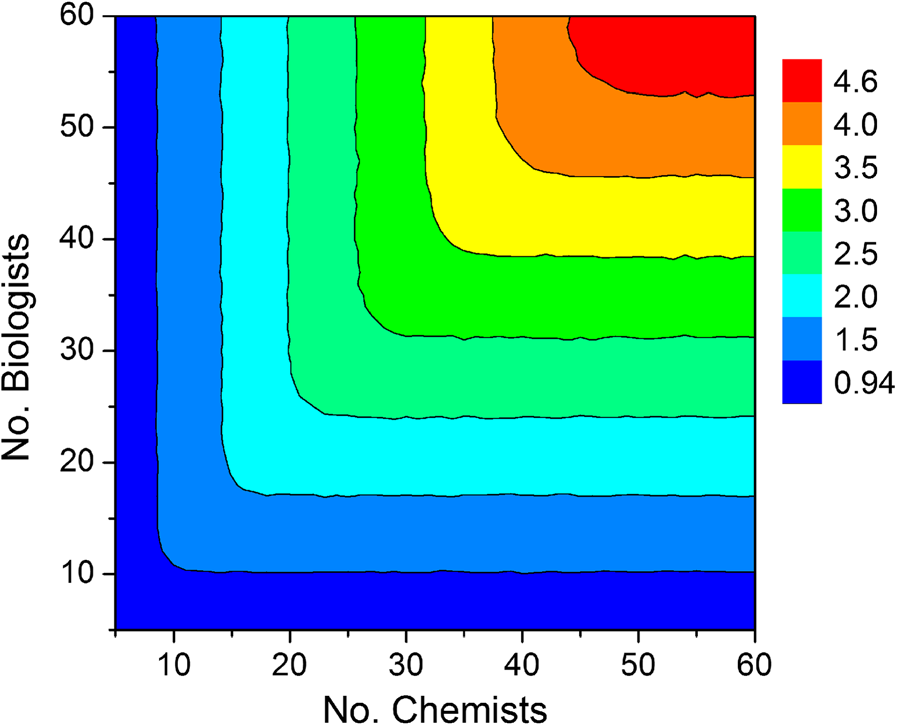
Relationship between number of chemists, number of biologists and output defined by the average number of preclinical candidate compounds per year.

In the absence of appropriate constraints, however, there would be an unbounded linear relationship between preclinical drug candidate output and group size, which could both artificially approach infinity in a linear fashion (Figure 4). Published studies, [11,12] anecdotal evidence, [13] and intuition, on the other hand, do not support the possibility of infinite growth as either realistic or even desirable, particularly with regard to early discovery (e.g., project ideation) and decision-making [14,15] While at its most basic, Condorset’s jury theorem might suggest that as group size approaches infinity, the probability of a correct decision (e.g., go/no-go milestone decision) approaches one given ρ is greater than 0.5 [16,17]. However, the theorem assumes that all members cast their vote independent of one another with uniform probabilities. Although the theorem has since been generalized, [18] mutual independence would clearly not be an optimal arrangement for scientists either within a cross-functional project team or in the larger working group charged with such decisions. Typically, in advance of the actual decision-making event reports must be prepared and meetings held in order to communicate results and ensure adequate understanding of the key issues. *Here the objective is a consensus decision through opinion aggregation*, [19]*rather than a simple majority vote*. Thus, as group size increases FTE “overhead” must also increase due to the exponential rise in the amount of correspondence, number of progress reports that must be written and read as well as the need for greater managerial oversight that may include multiple lines of reporting, especially in the case of global discovery project teams [20]. In large pharmaceutical companies, for example, scientists are typically subdivided into cross-discipline specialty centers, units, departments or divisions to create more manageable, functional sized, and focused working groups. A correction factor was therefore needed to mathematically relate productivity to FTE efficiency as a function of working group size. Paramount to this is the ability of working group members to make the most informed decisions in a timely manner, [21,22] since that lies at the heart of project progression through a drug discovery milestone system.

Unfortunately, publications in this area primarily address single, relatively small teams of less than 10–20 people that do not interact with other teams as part of a larger working unit [23] We therefore needed a general way to quantitate efficiency as a function of group size in the context of multiple project teams. To accomplish this, we propose using a modified equation based on the diffusion of innovations theory popularized by Rogers, [24] which posits that individuals progress through five stages before adopting a new innovation or technology: knowledge, persuasion, decision, implementation, and confirmation. If we assume that a research group constitutes a type of social system, then communication in its many forms represents a critical factor that influences how new ideas become reduced to practice and recognized as a formal project through formation of a discovery project team. Decision-making becomes increasingly difficult, time-consuming and complex as working group size increases, particularly if there is a range of expertise in the group. In that sense, efficiency as defined by time spent on specific project related activities must be inversely proportional to the amount of time discovery project teams must spend dealing with bureaucracy [25] in its many forms. Just as diffusion of innovations follows a logistic curve, we propose that FTE efficiency as a function of working group size must follow a similar path, but with a positive Euler’s exponent (Equation 3 where x is group size, E(x) is efficiency as a function of group size, and parameters A and B define the decay rate and inflection point, respectively). In this manner, efficiency exponentially decreases as working group size increases, asymptotically approaching zero as group size approaches infinity.

(3) $$\text{E}\left(x\right)=\frac{1}{1+e^{\left(\frac{x}{A}-B\right)}}$$

where E is FTE efficiency, x is group size, A and B are positive, non-zero parameters.

From Equation 3, efficiency equals 0.5 when group size equals the product of parameters A and B. However, since A and B are abstract values and therefore difficult to assign a priori, another form of the equation was derived. Two alternative parameters, M and N, were defined as the group size with 50% and 75% FTE efficiency, respectively. Substituting into Equation 3, rearranging, and solving for parameter A yields Equation 4. The value for B was obtained by simple rearrangement. Substituting those values for A and B back into Equation 3 then affords Equation 5. The parameters M and N are under user control and can thus be adjusted depending on individual company culture, accepted communication norms, organizational structure (e.g., degree of globalization within the working group) and reliance on third-party outsourcing.

(4) $$\begin{array}{l} A\;=\frac{M-N}{1.1}\\ B\;=\frac{M}{A} \end{array}$$

where M = group size operating at 50% efficiency and N = group size operating at 75% efficiency. In all cases, M>N.

(5) $$\text{E}\left(\text{x}\right)=\frac{1}{1+{\text{e}}^{\left(\frac{1.1\left(\text{x}-\text{M}\right)}{\text{M}-\text{N}}\right)}}$$

Where x is group size and E(x) is FTE efficiency as a function of group size. M and N are defined in Equation 4.

The user now only needs to estimate the ratio of time spent by scientists on direct project related activities versus other responsibilities for a given group size in order to calculate the FTE efficiency curve. It is expected that these estimates would vary across companies or even across individual working groups operating within a larger organization.

The Monte Carlo simulation algorithm was written in C and compiled using a GNU C compiler for execution on a Windows platform (please contact the author for a copy of the source code and accompanying control file). At present there is no GUI associated with the computer program. Rather, simulation parameters and other control values are entered through a simple tab-delimited text file. At some future point, a front-end GUI may be constructed to make the program more user-friendly.

### Experimental

To run the program, the user must first enter control parameter values into a simple tab-delimited text file. Upon launch, the program reads the file whose parameters are defined below:

Probability of achieving Hit to Lead milestone

Probability that an exploratory effort will successfully (1) develop a multi-tiered evaluation system for compound screening against a validated biological target, and (2) identify active hit compounds (range 0–1).

Probability of achieving Lead Optimization milestone

Probability that a hit to lead project will successfully (1) identify an optimizable lead series of compounds, and (2) define a target drug profile (range 0–1).

Probability of achieving Preclinical Development milestone

Probability that a lead optimization campaign will successfully identify a preclinical candidate compound that satisfies the target drug profile criteria (range 0–1).

Percentage of follow-on Hit to Lead projects

Percentage of hit to lead projects that share a common biological target with another on-going project, but with a different scaffold or lead series (range 0–0.5).

Percentage of chemistry-driven Hit to Lead projects

Percentage of hit to lead projects that involve a specific compound or compound series whose MOA may or may not be known. The balance of hit to lead projects are assumed to have arisen through HTS involving a validated biological target (range 0–1).

Percentage of prioritized Lead Optimization projects

Percentage of lead optimization projects that will be designated as high priority. The balance of lead optimization projects will be assigned standard priority (range 0–1).

Target no. Hit to Lead CHEMISTS (chemistry-driven) per team

Target number of chemists that will be assigned to a chemistry-driven hit to lead project (integer>0).

Target no. Hit to Lead BIOLOGISTS (chemistry-driven) per team

Target number of biologists that will be assigned to a chemistry-driven hit to lead project (integer>0).

Target no. Hit to Lead CHEMISTS (biology-driven) per team

Target number of chemists that will be assigned to an HTS directed hit to lead project (integer>0).

Target no. Hit to Lead BIOLOGISTS (biology-driven) per team

Target number of biologists that will be assigned to an HTS directed hit to lead project (integer>0).

Target no. Hit to Lead CHEMISTS (follow-on) per team

Target number of chemists that will be assigned to a follow-on type of hit to lead project (integer>0).

Target no. Hit to Lead BIOLOGISTS (follow-on) per team

Target number of biologists that will be assigned to a follow-on type of hit to lead project (integer>0).

Target no. Lead Optimization CHEMISTS per team

Target number of chemists that will be assigned to a lead optimization project (integer>0).

Target no. Lead Optimization BIOLOGISTS per team

Target number of biologists that will be assigned to a lead optimization project (integer>0).

No. simultaneous lead optimization projects supported by DMPK

Maximum number of discovery projects that can be supported simultaneously by the DMPK group (integer≥0).

Percent impact on timeline by DMPK support

Percent shortening of timeline if DMPK support is present (range 0–1).

Target cycle time (months) to achieve Lead Optimization

Project cycle time from hit to lead to lead optimization (integer>0).

Target cycle time (months) to achieve Preclinical Development

Project cycle time from lead optimization to candidate selection (integer>0).

Group size at 75% FTE efficiency

Estimated working group size where each member spends approximately 75% of their time on direct project related activities. This will depend on company culture, corporate policies and how direct project related activities are defined by the user (integer>0).

Group size at 50% FTE efficiency

Estimated working group size where each member spends approximately 50% of their time on direct project related activities. This group size value must be greater than the one for 75% FTE efficiency (integer>0).

Additional parameters include those for defining the total number of chemists and biologists that comprise the working group to be simulated, the time period for data collection, and the number of replicate runs. The user also has the ability to override the FTE efficiency calculation and enter a static value (e.g., 1 for 100% FTE efficiency regardless of working group size). An example list of parameter values can be found in the Results and Discussion section.

Upon completion of the run, a text file is created that contains the predicted average number of preclinical candidates per year in a single column format. This can be converted to a matrix form using a variety of commercial software packages (e.g., OriginPro [26]) and the results displayed as either a two- or three-dimensional plot.

## Results and discussion

**Figure 5:**
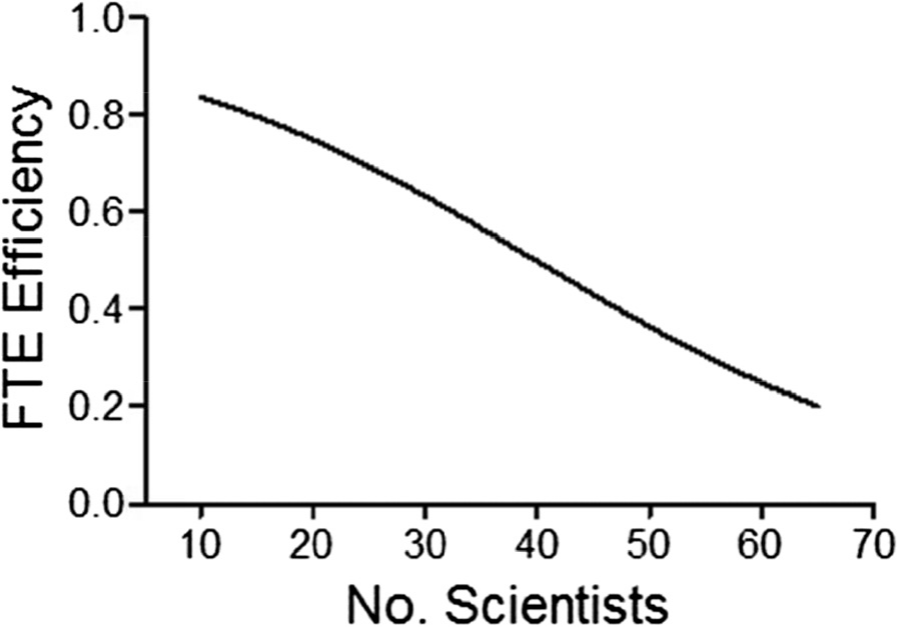
FTE efficiency curve generated by Equation 5 with M=40 and N=20.

A possible starting approximation is that FTE efficiency is 75% when the size of a single working group (i.e., department, division or specialty center) of scientists approaches 20 and may be projected to be 50% when that number reaches 40. Entering those starting estimates into Equation 5 as parameters N and M, respectively, affords the FTE efficiency curve shown in Figure 5.

**Figure 6:**
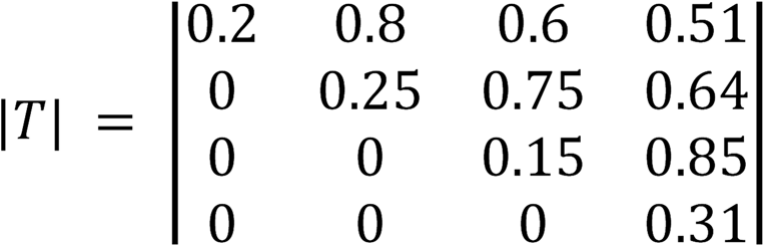
Transition probability matrix for the discovery milestone system using values reported by Paul and co-workers^1^.

**Figure 7:**
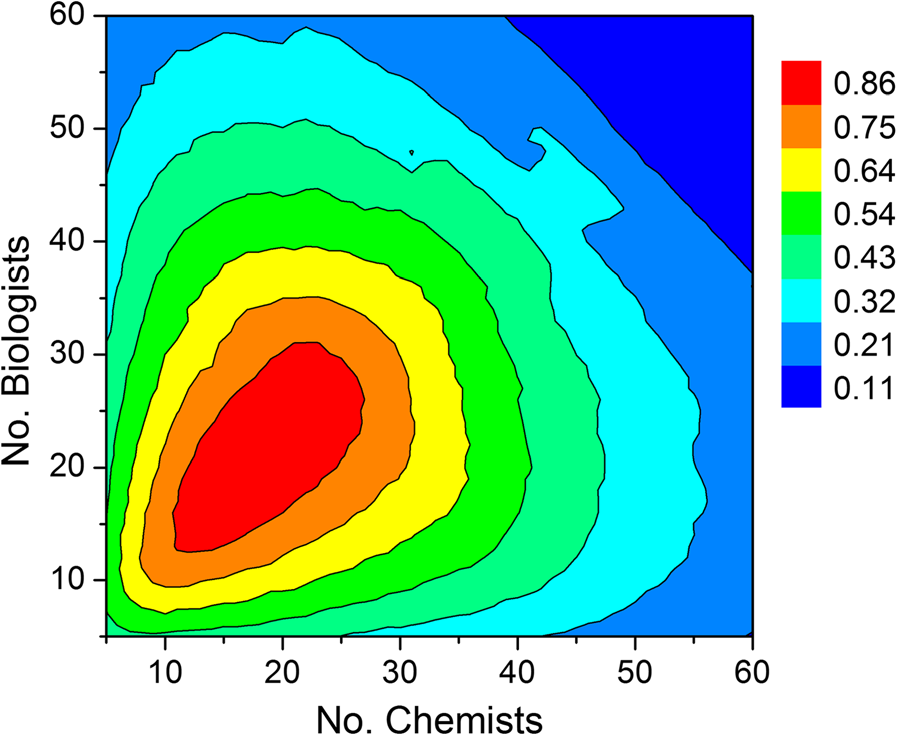
**Simulation results using the assumptions outlined in Table**1**.** Output values are the average number of preclinical candidate compounds generated per year for a given combination of chemists and biologists.

The percent technical success criteria reported by Paul and co-workers^1^ for compounds to reach the hit to lead, lead optimization, preclinical development and clinical development milestones are 80%, 75%, 85%, and 69% respectively. Thus, the milestone system with observed transition probabilities could be considered a simple Markov chain with a transition probability matrix as shown in Figure 6. Applying those values to percent project success thresholds along with the simulation parameters summarized in Table 1 provided the result illustrated in Figure 7.

**Table 1:** Parameters used in simulation^a^

Probability of achieving Hit to Lead milestone	0.80
Probability of achieving Lead Optimization milestone	0.75
Probability of achieving Preclinical Development milestone	0.85
Percentage of follow-on Hit to Lead projects	0.25
Percentage of chemistry-driven Hit to Lead projects	0.5
Percentage of prioritized Lead Optimization projects	0
Target no. Hit to Lead CHEMISTS (chemistry-driven) per team	2
Target no. Hit to Lead BIOLOGISTS (chemistry-driven) per team	3
Target no. Hit to Lead CHEMISTS (biology-driven) per team	1
Target no. Hit to Lead BIOLOGISTS (biology-driven) per team	3
Target no. Hit to Lead CHEMISTS (follow-on) per team	2
Target no. Hit to Lead BIOLOGISTS (follow-on) per team	1
Target no. Lead Optimization CHEMISTS per team	4
Target no. Lead Optimization BIOLOGISTS per team	4
No. simultaneous Lead Optimization projects supported by DMPK	0
Percent impact on timeline by DMPK support	0.1
Target cycle time (months) to achieve Lead Optimization	18
Target cycle time (months) to achieve Preclinical Development	24
Group size at 75% FTE efficiency	20
Group size at 50% FTE efficiency	40

^a^See Experimental section for a detailed explanation of the control variables.

Under these assumptions, the optimum total number of scientists in a working group is predicted by the model to be around 36 (18 chemists, 18 biologists and sufficient DMPK support to allow decisions to be made within the published cycle times) with an expected average output of 0.86 preclinical candidates per year. The pharmaceutical R&D productivity model proposed by Paul and coworkers^1^ indicates that approximately 11 small molecule compounds must enter clinical development to afford 1 NME launch every year. Taken together, our Monte Carlo model predicts that an organization aspiring to produce a minimum of 1 NME launch per year would need 12–13 working groups of that size or its equivalent output in the form of strategic alliances, mergers or acquisitions to meet its annual productivity objective. While these numbers would be expected to vary across companies, the modeling results do allow users within a company to assess the impact that project portfolio as well as working group size might have on expected output. Since these projections are a function of the simulation parameters specified by the user, they will change depending on the values entered.

In this regard, the Monte Carlo model would support “what if” scenarios to be tested with varying input parameters, thereby revealing potential areas of opportunity for either improvement or optimization. Too many preclinical candidates in a given time-frame could potentially stress development resources and thereby increase downstream cycle times. This, in turn, would ultimately adversely affect overall productivity of the company in terms of NME launches per unit time [27]. Too few and gaps appear in the clinical development pipeline that cannot be filled by internal research and must be addressed through external means such as in-licensing or joint ventures. Predicted value and probability of technical success for the preclinical candidates that emerge from the discovery pipeline are critically important, certainly more so than simply the absolute numbers that emerge. While the model does not explicitly take these particular parameters into account, the assumption is made that the working group conducts those evaluations as part of the decision-making process as reflected in the attrition rates.

**Figure 8:**
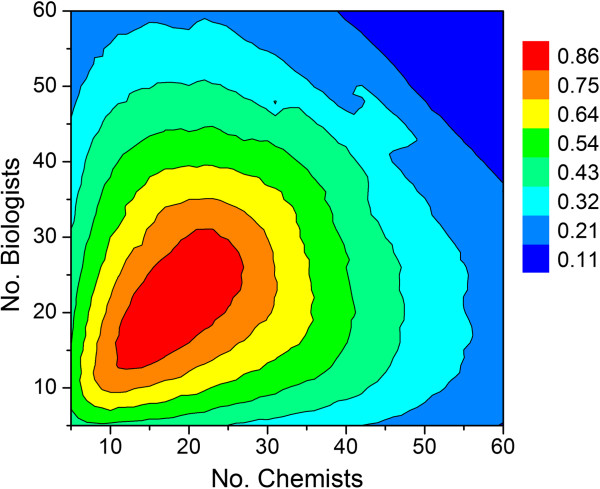
**Simulation results using the assumptions listed in Table**1**, except all projects at the lead optimization stage were given high priority.** Output values are average number of preclinical candidate compounds generated per year for a given combination of chemists and biologists.

Inspection of Figure 7 indicates that growing a single working group size beyond approximately 36 scientists does not lead to a commensurate increase in productivity. In fact, given the simulation assumptions listed in Table 1, the maximum output per working group is predicted to be 0.86 preclinical candidates per year, which is a direct consequence of the FTE efficiency correction factor. This particular simulation assumed that all lead optimization projects were given standard priority. If all projects at the lead optimization stage were given high priority, then the predicted number of preclinical candidates per year is predicted to drop by one third (Figure 8). The simulation therefore suggests that terminating earlier projects to support a high priority lead optimization campaign may help achieve short-term objectives, but consistently doing so will have a deleterious effect on long term productivity. While this may not be preferable in all instances, sacrificing longer term productivity to achieve short-term objectives may be appropriate under certain circumstances. For example, if there are first-in-class drug candidates with an unproven MOA, or there are too many clinical candidates competing for development resources, then it may be necessary to review the milestone progression criteria and adjust them accordingly with an eye toward achieving clinical POC as soon as possible.

**Figure 9:**
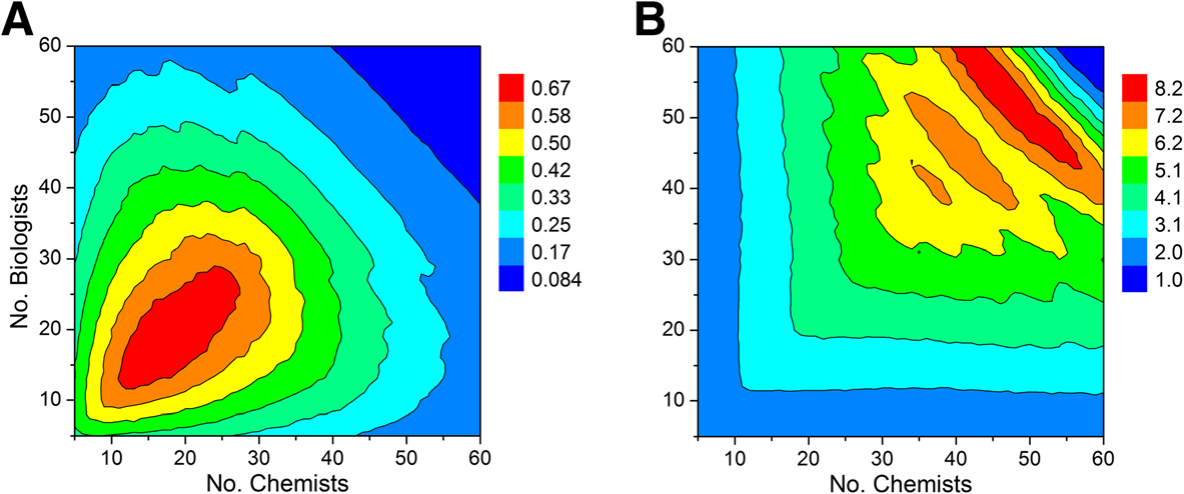
**Contour plot showing relationship between number of chemists, biologists and predicted output for a 1:1 ratio of high priority and standard priority lead optimization campaigns.** See Table 1 for all other assumptions. (**A**) Output defined by the average number of preclinical candidates per year. (**B**) Output defined by the number of active lead optimization projects per year.

**Figure 10:**
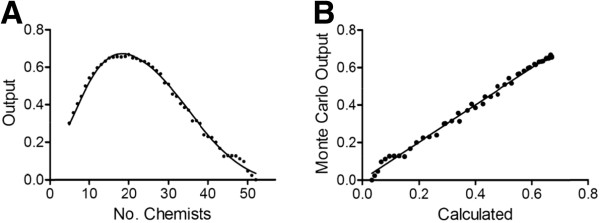
**(A) Monte Carlo simulation output values extracted from the diagonal of Figure**8**(points).** Output from Equation 6 (solid line). Under the simulation conditions, maximum output occurs with 18 chemists. (**B**) Output from Equation 6 versus Monte Carlo simulation output values extracted from the diagonal of Figure 8 with linear regression line (r^2^ = 0.99).

Assuming a 1:1 ratio of standard and high priority lead optimization campaigns along with the assumptions listed in Table 1, the model predicts the maximum average number of preclinical candidates per year to be around 0.67 (Figure 9). The diagonal leading edge of the contour in Figure 9A marks the optimum combination of chemists and biologists for a given portfolio of drug discovery programs, cycle times and project team sizes. Extracting the values along the diagonal and plotting output (average number of preclinical candidates per year) as a function of the number of chemists affords a biphasic curve [28] that can be empirically modeled using Equation 6 (Figure 10). As before, maximum output occurs with 18 chemists. Thus, for a given set of assumptions, the impact of chemistry (or biology) group size can be assessed in terms of overall predicted productivity. Whether the analysis is chemistry-centric or biology-centric will depend on the type of projects under consideration. For example, projects targeting a best-in-class drug candidate may need little exploratory biology and strong chemistry support, whereas those targeting a first-in-class drug may need extensive exploratory biology and a small, but long-term chemistry commitment since relatively little may be known to connect MOA with a human disease state. Similarly, a total synthesis program involving a structurally complex chemical lead may require a critical mass of chemists at the outset just to get off the ground.

(6) $$\text{y}=\text{t}-\frac{\text{Aup}}{1+{\text{Bup}\cdot \text{e}}^{\left(\frac{\text{x}}{\text{Cup}}-\text{Dup}\right)}}-\frac{\text{Adn}}{1+{\text{Bdn}\cdot \text{e}}^{\left(\frac{-\text{x}}{\text{Cdn}}+\text{Ddn}\right)}})$$

Where:

x = number of chemists

y = predicted average number of preclinical candidates per year

t ≈ 0.802

Aup ≈ 0.798

Adn ≈ 0.849

Bup ≈ 1.971

Bdn ≈ 0.219

Cup ≈ 3.996

Cdn ≈ 7.610

Dup ≈ 2.402

Ddn ≈ 6.093

**Figure 11:**
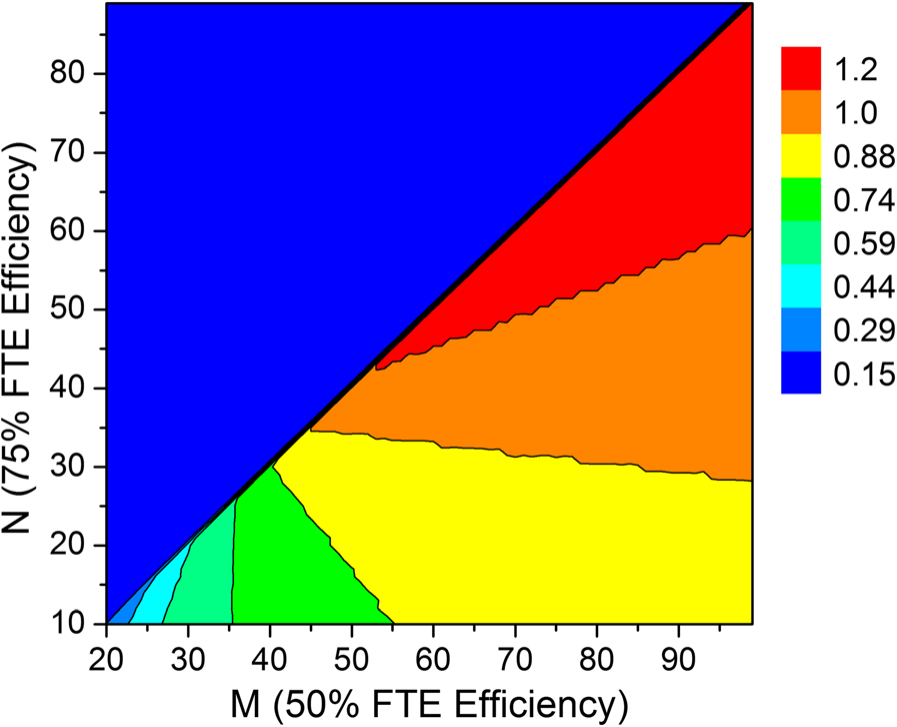
Relationship between FTE efficiency parameters M, N and predicted average number of preclinical candidates per year for a working group of 36 scientists (18 chemists and 18 biologists) and 1:1 ratio of high priority to standard priority lead optimization projects.

The relationship between FTE efficiency and predicted output for a group of 36 scientists divided equally between chemists and biologists was investigated next, again assuming a 1:1 ratio of standard to high priority lead optimization projects. In this simulation, the values of M and N were systematically varied with the limitation that M must be greater than or equal to N+10. All other parameters listed in Table 1 were held constant. As expected, productivity is dependent on FTE efficiency. For example, if FTE efficiency is 75% with a group size of 20–30 and 50% with a group size of 55–65, then the maximum average output is predicted to be around 0.88 preclinical candidates per year (Figure 11). If parameters M and N are sufficiently large, i.e., FTE efficiency is sufficiently high, then under the simulation conditions the maximum average output could reach 1.2 preclinical candidates per year.

Inspection of Equation 1 indicates that project timelines (i.e. time to next milestone) are also affected by FTE efficiency. As defined by the algorithm, two scientists working at 50% FTE efficiency are equivalent to one scientist working at 100% FTE efficiency. Thus, efficiency and level of staffing work hand-in-hand by the model to define the time to next milestone. Inspection of the simulation output (data not shown) indicates that at either low efficiency or low staffing the time to next milestone can become unacceptably long. Consequently, the algorithm was modified such that projects that have an elapsed time longer than a user-specified time period are simply terminated and the scientists reassigned to other projects according to the priority outlined in Figure 3.

**Figure 12:**
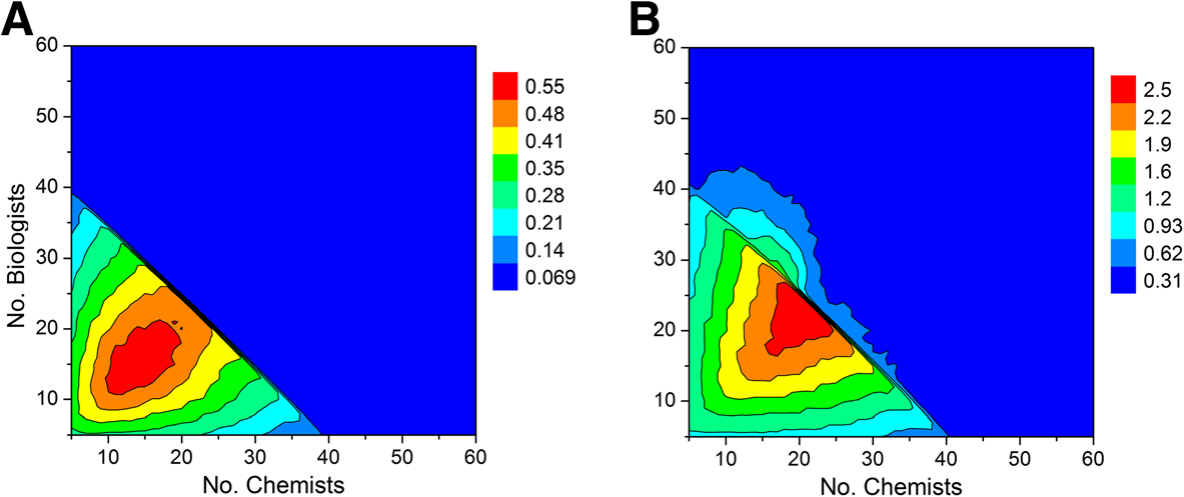
**Contour plot showing relationship between number of chemists, biologists and predicted output for a 1:1 ratio of high priority and standard priority lead optimization campaigns.** Cut-off values of 3.5 and 4.5 years were applied for hit to lead and lead optimization activities, respectively, after which the projects were simply terminated. See Table 1 for all other assumptions. (**A**) Output as defined by the average number of preclinical candidates per year. (**B**) Output defined by the average number of active lead optimization projects per year.

Repeating the simulation using cut-off values of 3.5 and 4.5 years for hit to lead and lead optimization activities, respectively, with the set of assumptions described above yields the contour plots shown in Figure 12. In addition to a slightly diminished maximum output (0.55 vs. 0.67), there is a sharp, seemingly paradoxical decline in productivity that occurs at large group sizes (cf. Figures 9A and 12A). This is due to the longer project timelines associated with decreased FTE efficiency. If efficiency drops to a sufficiently low level, then many of the lead optimization projects will reach their time limit before the next milestone go/no-go decision. This effect becomes very clear when a hard cut-off filter is applied that restricts the lifetime for hit to lead and lead optimization campaigns. Taken together, the model predicts that there is an optimum working group size beyond which productivity will either remain flat or actually decline in tandem with the number of viable lead optimization campaigns. This number appears to be unaffected by the priority given to lead optimization projects, which under conditions of the simulation is less than 40 scientists. Increasing group size above this value will lead to a proportional decrease in FTE efficiency, which in turn will lead to lower productivity. In an attempt to off-set the effect of low efficiency, the tendency of individual project teams might be to seek an increase in membership. If granted, however, this would lead to fewer projects the working group can support, thereby resulting in fewer preclinical candidates to ultimately emerge from the discovery pipeline.

**Figure 13:**
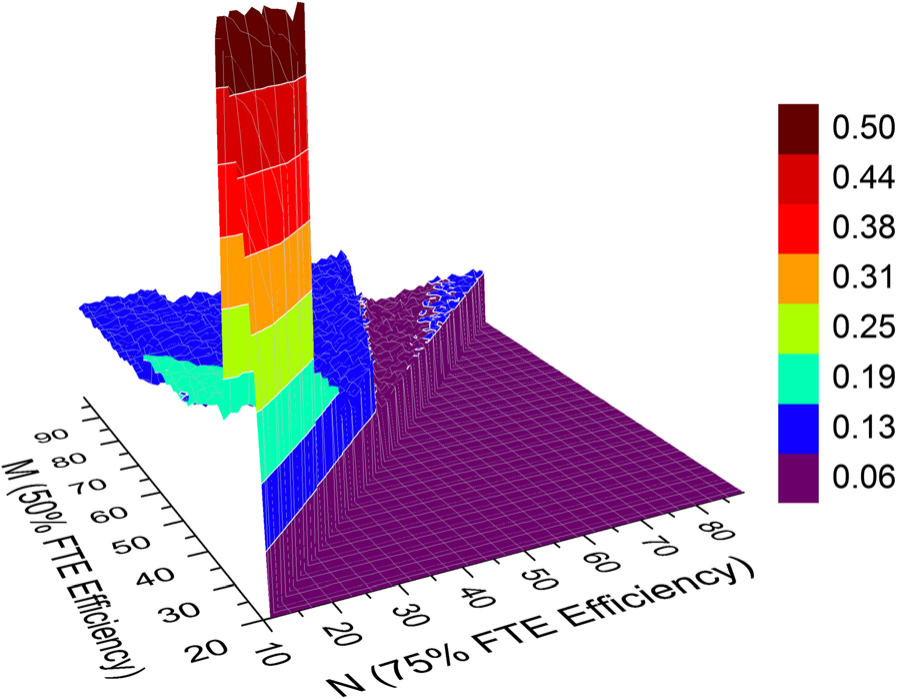
**Productivity difference map between two FTE efficiency simulations that systematically varied M and N for 18 chemists and 18 biologists (1:1 ratio of high priority to standard priority lead optimization projects).** The only difference between the two runs is the presence or absence of project lifetime cut-off values applied for hit to lead and lead optimization activities (3.5 and 4.5 years, respectively). See Table 1 for all other assumptions. The values along the z-axis represent the difference in predicted productivity output between the two simulations, where productivity is slightly higher in the absence of timeline constraints.

Repeating the FTE efficiency simulation (see Figure 11) with 36 scientists as described earlier, but using lifetime cut-off values of 3.5 and 4.5 years for hit to lead and lead optimization campaigns, respectively, afforded very similar results where the maximum output in both cases is predicted to be around 1 preclinical candidate per year on average. However, differences were noted, particularly at lower FTE efficiency (Figure 13). Smaller values of M and N lengthen project timelines, thereby giving rise to campaigns that run the risk of premature termination. In this regard, very little differences were noted at the higher FTE efficiency range (i.e., larger values of M and N).

**Figure 14:**
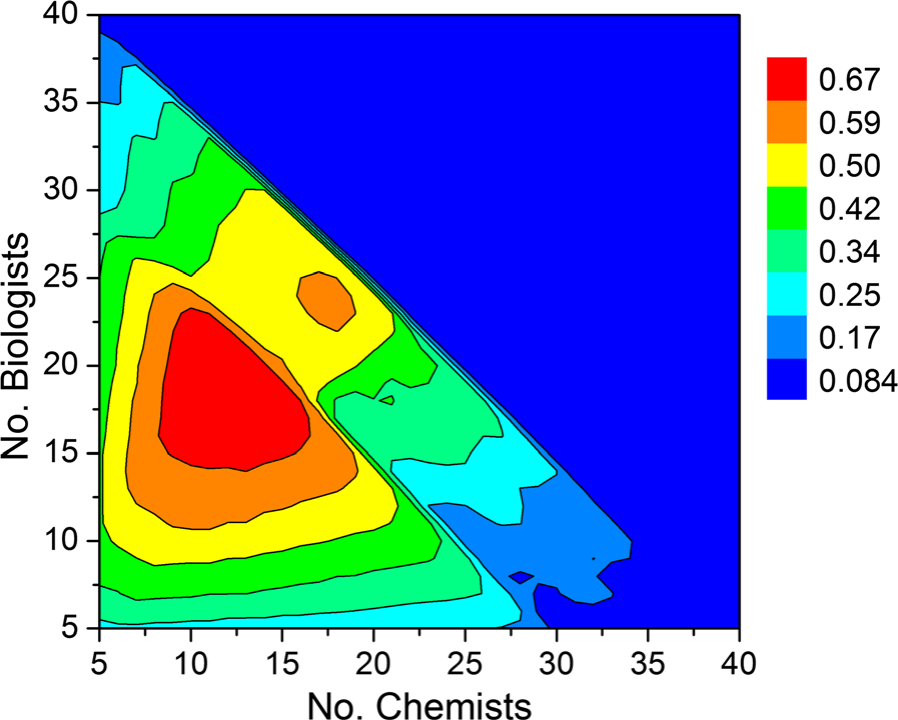
**Contour plot showing relationship between number of chemists, biologists and predicted preclinical candidate output for biology-driven projects.** Cut-off values of 3.5 and 4.5 years were applied for hit to lead and lead optimization activities, respectively, after which the projects were simply terminated. See Table 1 for all other assumptions.

Modifying the conditions of the simulation whereby only certain types of projects are undertaken allows the ratio of chemists to biologists to be assessed. As expected, the optimal ratio of biologists to chemists was shifted upward when projects that require greater exploratory biology were created (Figure 14). Exceeding the optimal number of chemists in the group did not lead to greater output since under those conditions projects became biology rate-limited. Conversely, for projects that require greater chemistry support adding more biologists to the working group would not lead to an increase in productivity as projects in that case would become chemistry rate-limited. Thus, for a given ratio of chemists and biologists there is an optimum combination of project types that can be supported.

**Figure 15:**
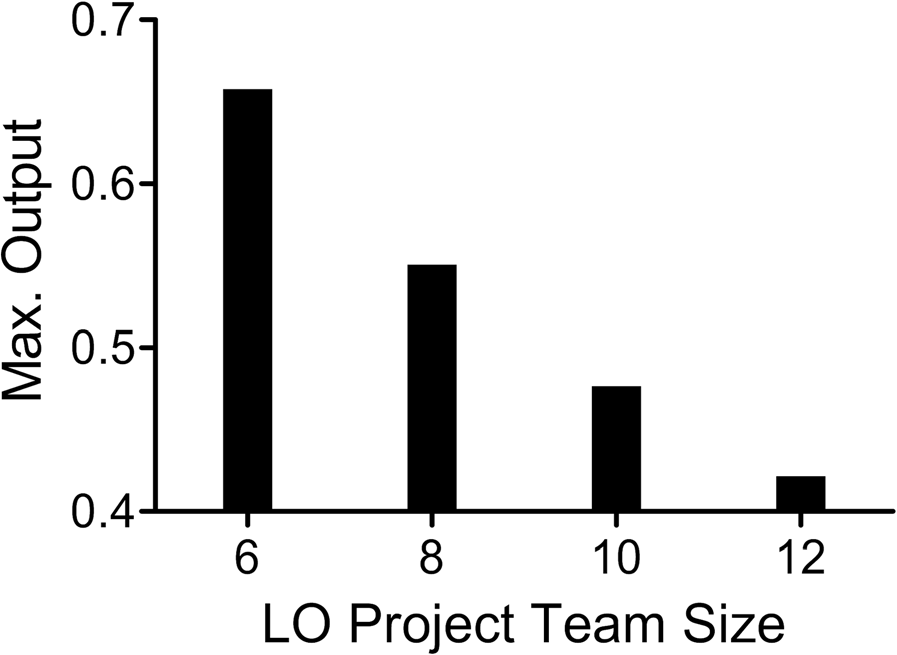
**Relationship between lead optimization project team size and maximum number of preclinical candidates produced by a working group of 10–40 scientists.** See text and Table 1 for simulation parameters.

Project team size requirements can exert a significant impact on productivity. As team size grows, project numbers shrink, resulting in fewer preclinical candidates to emerge from the discovery pipeline. As an example, the size of lead optimization project teams was varied from 6 to 12 scientists, equally split between chemists and biologists. As before, a 1:1 ratio of high priority to standard priority was assumed along with assigning M=40 and N=20. All other parameters were held constant (Table 1). In this simulation, the working group size was allowed to span the range of 10–40 scientists and the maximum predicted output in terms of preclinical candidates was captured. As illustrated in Figure 15, there is a clear decline in maximum productivity as project team size requirements grow.

**Figure 16:**
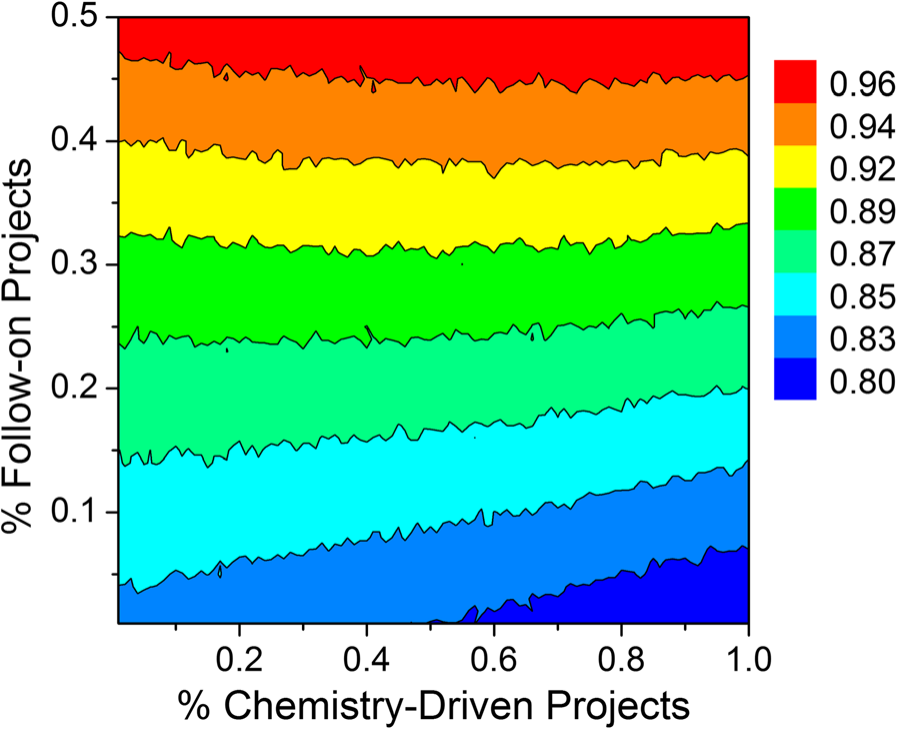
**Relationship between percent chemistry-driven projects versus percent follow-on projects in terms of preclinical candidate output for a working group of 18 chemists and 18 biologists.** See text and Table 1 for simulation parameters.

The types of projects undertaken by a working group can also affect output, albeit to a much lesser extent than project team size. Assuming a 1:1 ratio of high priority to standard priority lead optimization projects, a fixed FTE efficiency of 80% and the simulation parameters listed in Table 1, the output for 36 scientists (18 chemists and 18 biologists) as a function of percent follow-on projects versus percent chemistry/biology driven projects was predicted (Figure 16). Higher output was noted when a larger percentage of follow-on projects was undertaken. In this analysis, little if any differences were noted when varying the percentage of chemistry driven projects.

**Figure 17:**
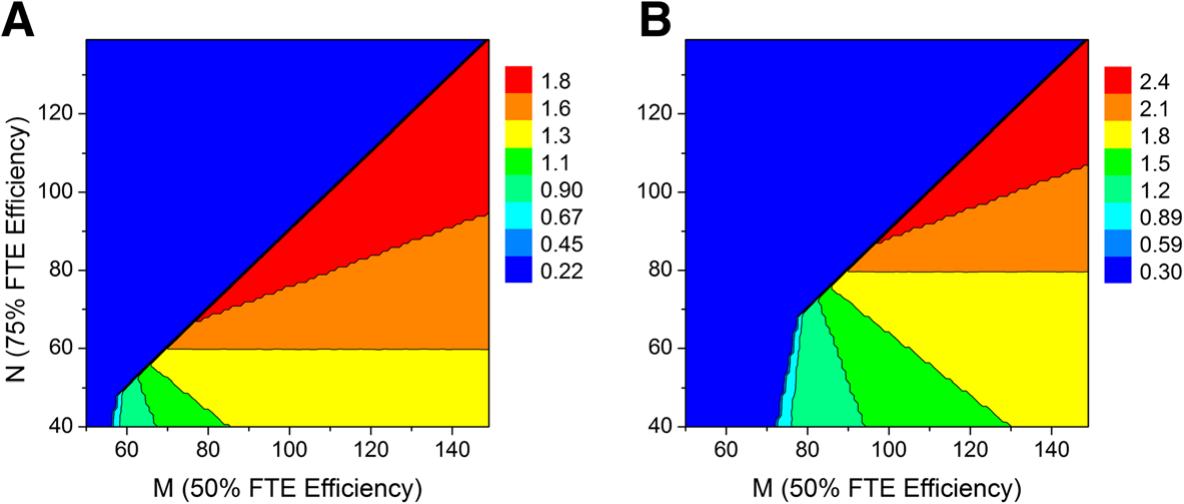
**Relationship between FTE efficiency parameters M, N and predicted average number of preclinical candidates per year (1:1 ratio of high priority to standard priority lead optimization projects with hit to lead and lead optimization project lifetime cutoff values of 3.5 and 4.5 years, respectively.** (**A**) Working group of 60 scientists (30 chemists and 30 biologists). (**B**) Working group of 80 scientists (40 chemists and 40 biologists).

The FTE efficiency simulation with two relatively large working groups consisting of 60 and 80 scientists again equally split between chemists and biologists was run to afford the results shown in Figure 17. At sufficiently high FTE efficiency, the maximum output predicted for these two particular groups is 1.9 and 2.5 preclinical candidates per year, respectively. Based on these results, the next series of simulations were run with the FTE efficiency parameters N and M set to 100 and 150, respectively.

**Figure 18:**
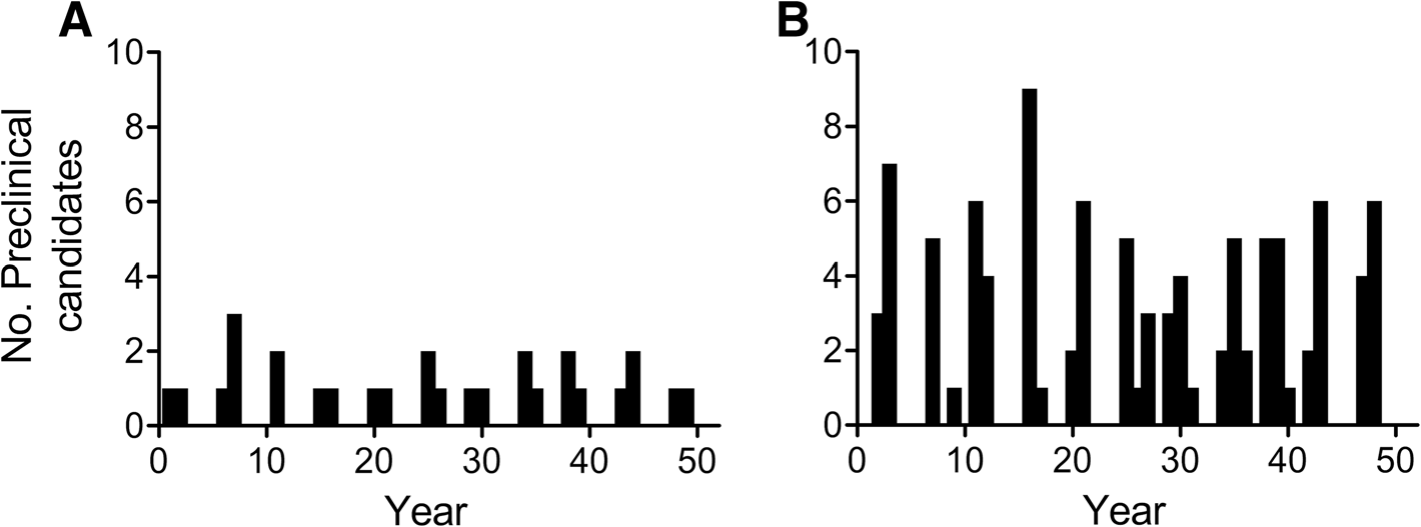
**Time chart showing simulated preclinical candidate output as a function of year for a working group comprised of the following: (A) 10 chemists and 10 biologists with an average output of 0.6. **(**B**) 40 chemists and 40 biologists with an average output around 2.

To evaluate the number of preclinical candidates to emerge in unit time, two simulations were run with different sized working groups under the set of assumptions described above. For a relatively small working group of 20 scientists the output is predicted to be 0.6 preclinical candidates per year on average. For a larger group of 80 scientists the predicted output is around 2. Plotting number of preclinical candidates as a function of time for both working groups reveals that compounds are predicted to pass the candidate selection milestone and enter preclinical development at irregular intervals (Figure 18). This appears to be independent of working group size. For the 20 and 80 member groups, the number of preclinical candidates in any given year is predicted to range from 0–3 and 0–9, respectively. The wide variation in both cases suggests that working groups, regardless of size, can experience prolonged trough periods of low apparent output followed by periods of high productivity as measured by the number of preclinical candidates to emerge from the discovery pipeline. This is likely a consequence of projects moving at different speeds through the milestone system as a result of different project priorities and resource support limitations. The model therefore predicts that careful project management will be necessary to match discovery output over time with downstream development resource capacity.

A careful analysis and evaluation of working group size, project team size, cycle time and FTE efficiency are important steps to better understand the differences, if any, that may exist between current output and performance expectations. For example, if simulations suggest that a target output of 2 preclinical candidates per year for a particular working group might not be realistic even under optimal conditions for a specific set of assumptions, then the team may wish to consider ways to enhance production that may involve increasing working group size or changing resource deployment strategies. Since the objective of drug discovery is to feed the drug development pipeline and achieve clinical POC as soon as possible, then simulations may help define where specific discovery milestones should be placed relative to each other. This will undoubtedly impact transition probabilities and redefine resource needs depending on the type of programs undertaken in connection with the working group’s strategic plan. Other opportunities may include resource sharing with other working groups to identify compounds with a similar MOA, but different disease targets or it could involve internal or external collaborations that take advantage of specific enabling technologies. An alternative to expanding a single working group might be to create and grow a separate working group altogether. This would obviate much of the FTE overhead associated with a large group, but still allow overall productivity to increase commensurate with the number of scientists.

To benchmark working group performance, the absolute maximum output for a group of 18 chemists and 18 biologists operating with an FTE efficiency of 100% and all milestone transition probabilities set to 100% was simulated. In this experiment, all lead optimization projects received full DMPK support, and a maximum lifetime of 3.5 and 4.5 years for hit to lead and lead optimization projects, respectively, was used. All other parameters were set to those listed in Table 1. Under these conditions, the model predicts that a maximum of 2 preclinical candidates per year would be generated by such a department, division or specialty center. The average output for an identical sized working group, but operating with a fixed FTE efficiency of 80% and all milestone transition probabilities set to those reported by Paul and co-workers^1^ is predicted to be 1 preclinical candidate per year. By assuming that all lead optimization projects have high priority, the output drops slightly to 0.8. On the other hand, if all projects are mandated to have an on-going shared resource “back-up” series, then output is predicted to increase somewhat to 1.3 preclinical candidates per year. Thus, the model allows working group productivity to be assessed in the context of multiple variables, including FTE efficiency, project priority handling, types of projects, and milestone transition probabilities, factors that are closely tied to the working group’s overall research strategy. If discovery DMPK support becomes rate-limiting then productivity will incrementally decrease depending on the number of lead optimization projects that can simultaneously be supported.

## Conclusion

The productivity challenge facing the pharmaceutical industry is complex and multifactorial. While early discovery marks just the beginning of a very long and costly process of bringing an NME to market, it plays a critical role in helping to maintain a robust clinical development pipeline. Obviously, without preclinical candidates there would be no clinical POC studies. Successfully refocusing effort and resources earlier in the drug development process will require a strong portfolio of quality preclinical candidates that have a reasonable probability of technical success. In that regard, the Monte Carlo simulation suggests that attempting to improve drug discovery productivity by simply increasing the size of existing working groups may not necessarily be the best solution. On the contrary, the model predicts that for a given portfolio of discovery projects there is an optimum number of scientists, beyond which productivity in terms of preclinical candidates will remain flat. In such circumstances, establishing and growing a separate working group (i.e. a separate business unit, division or therapeutic specialty center) may be a more effective strategy, consistent with the concept of decentralized decision-making. For example, by creating business units that function more like biotech companies in terms of size, autonomy and accountability, but with the infrastructure and support of a large organization, FTE overhead associated with large groups may be substantially reduced. While there are many factors that impact how discovery projects progress through a milestone system, working group size and FTE efficiency are among the most amenable to change in order to optimize overall performance. Simulations also predict that the frequency of compounds to successfully pass the candidate selection milestone as a function of time will be irregular, with projects entering preclinical development in clusters marked by periods of low apparent productivity. Thus, the model may be useful as a tool to facilitate analysis of historical growth and achievement over time, help gauge current working group progress against future performance expectations, and provide the basis for dialogue regarding working group best practices and resource deployment strategies.
